# Skiascopy and Its General Application to the Study of Refraction

**Published:** 1895-11

**Authors:** 


SKIASCOPY AND ITS GENERAL APPLICATION TO THE STUDY OF REFRACTION. By EDWARD JACKSON, A. M., M. D., Professor of Diseases of the Eye in the Philadelphia Polyclinic and College for Graduates in Medicine ; Surgeon to the Wills Eye Hospital ; Chairman of the Section of Ophthalmology of the American Medical Association ; Member of the American Ophthalmological Society, etc. Twenty-six illustrations ; 8vo, pp. 112. Price, $1.00. Philadelphia : The Edwards & Docker Co. 1895.

Of all objective tests for errors of refraction of the eye, the shadow test, or skiascopy, is the most certain in its results, its application the most extended and its practice the most easily acquired. Dr. Jackson, with that conscientiousness and care which characterises all he does, has here given the best exposition of the subject that has yet been presented. The following synopsis will enable the reader to judge as to the contents of the book : Chapter I., history, name, difficulties and how to study the test ; Chapter II., general optical principles, reversal, real and apparent movement of light, rapidity of movement, form and brilliancy of light area, the point of reversal ; Chapter III., conditions of accuracy, source of light, focussing on retina, positions of accuracy, irregularities in media or surfaces ; Chapter IV., regular astigmatism, points of reversal, band-like appearance, changes with distance, direction of band and movements ; Chapter V., aberration and irregular astigmatism, the visual zone, symmetrical aberration, positive and negative, irregular astigmatism, conical cornea, the scissors movement ; Chapter VI., practical application with plane mirror, position and arrangement of light, H., E., M., regular astigmatism, aberration and irregular astigmatism, measurement of accommodation ; Chapter VII., practical application with concave mirror, position and arrangement of light, H., E., M., regular astigmatism, aberration and irregular astigmatism, measurement of accommodation ; Chapter VIII., general considerations, apparatus, mydriatics, relative advantages of plane and concave mirrors.

We heartily commend this little treatise to all ophthalmic practitioners, and trust that it will be instrumental in extending the use of this means of diagnosis, which to us has been invaluable for many years. The shadow-test should be resorted to more frequently than it is, and the instructions given by Dr. Jackson will enable any careful oculist to practise it intelligently and with satisfaction.

